# Toxicities of Fruquintinib in Gastrointestinal Malignancies: A Systematic Review and Meta-Analysis

**DOI:** 10.32604/or.2026.078524

**Published:** 2026-09-14

**Authors:** Daniel Thomas Jones, Tajveer Sangha, Arman Manjikian, Micheal Ghobrial, Qasim Shawesh, Emaan Tiwana, Emi Hearn, Arash Latif, Elaine Tupas, Aishwarya Hanspal, Rishi Kumar Nanda, Ramaditya Srinivasmurthy, Jason Ta, Meghana Pandit, Charles Abraham Joseph Larson, Kyaw Zin Thein

**Affiliations:** 1 Department of Internal Medicine, Sunrise Health GME Consortium, Las Vegas, NV, USA; 2 College of Osteopathic Medicine, Touro University Nevada, Las Vegas, NV, USA; 3 Department of Internal Medicine, Mount Sinai Morningside/West, New York, NY, USA; 4 Department of Internal Medicine, HCA Healthcare/USF Morsani College of Medicine GME Consortium, HCA Florida Citrus Hospital, Inverness, FL, USA; 5 Trinity School of Medicine, Warner Robins, GA, USA; 6 Division of Hematology and Medical Oncology, Comprehensive Cancer Centers of Nevada, Central Valley, Las Vegas, NV, USA

**Keywords:** Fruquintinib, gastrointestinal malignancies, metastatic colorectal cancer, vascular endothelial growth factor receptor, tyrosine kinase inhibitors, meta-analysis

## Abstract

**Backgrounds:** Fruquintinib is a selective vascular endothelial growth factor receptor (VEGFR)-1/2/3 inhibitor approved for previously treated metastatic colorectal cancer. As its use expands across gastrointestinal (GI) malignancies and combination regimens, randomized evidence is needed to define the toxicity profile most relevant to clinical monitoring, particularly hypertension, dermatologic toxicity, renal toxicity, bleeding, and thrombotic events. The objective of this study was to synthesize randomized controlled trial evidence to quantify the incidence and relative risk of key toxicities associated with fruquintinib in gastrointestinal malignancies. **Methods:** MEDLINE, EMBASE, and Cochrane CENTRAL were searched from inception through 1 January 2026 for phase II–III randomized controlled trials of fruquintinib in GI cancers reporting hypertension, proteinuria, hemorrhage, venous thromboembolism (VTE), and hand-foot skin reaction or palmar-plantar erythrodysesthesia (HFSR/PPE). Trial-reported CTCAE adverse events were pooled as risk ratios (RRs) with 95% confidence intervals (CIs) using Mantel-Haenszel random-effects models. Exploratory subgroup analyses were performed in metastatic colorectal cancer (mCRC). **Results:** Four randomized trials (n = 1872) were included. Fruquintinib significantly increased grade ≥3 hypertension (RR 9.01, 95% CI 4.67–17.40) and grade ≥3 HFSR/PPE (RR 26.00, 95% CI 6.42–105.29). Any-grade proteinuria was also increased (RR 1.89, 95% CI 1.30–2.74). Grade ≥3 hemorrhage occurred at low absolute rates and was numerically higher with fruquintinib, but the pooled estimate did not reach statistical significance (RR 1.82, 95% CI 0.92–3.61). VTE estimates were imprecise because of sparse events (any-grade VTE: RR 1.23, 95% CI 0.53–2.88; grade ≥3 VTE: RR 1.96, 95% CI 0.52–7.36). **Conclusions:** In randomized evidence across GI malignancies, the principal safety signals associated with fruquintinib are grade ≥3 hypertension and grade ≥3 HFSR/PPE, together with increased any-grade proteinuria. Grade ≥3 hemorrhage was uncommon and numerically higher, whereas VTE estimates remained imprecise. These findings support early blood pressure optimization, proactive dermatologic management, routine urinalysis monitoring, and individualized assessment of bleeding and thrombotic risk when initiating therapy.

## 1 Introduction

Gastrointestinal malignancies remain a leading cause of cancer-related morbidity and mortality worldwide, with particularly poor outcomes in advanced and refractory disease [1,2]. In metastatic colorectal cancer, progression after fluoropyrimidine-, oxaliplatin-, and irinotecan-based chemotherapy, together with prior biologic therapy, defines a later-line setting characterized by diminishing clinical benefit and increasingly limited treatment tolerability [1,2]. Similar challenges are encountered in advanced gastric and gastroesophageal junction cancers, where disease-related frailty and cumulative treatment toxicity frequently restrict salvage therapeutic options [3]. Therapeutic decision-making depends not only on antitumor efficacy but also on a clear understanding of treatment-related toxicities that may limit the ability to sustain effective therapy.

Angiogenesis is a central component of the tumor microenvironment and a critical driver of tumor growth, invasion, and therapeutic resistance in gastrointestinal cancers. Vascular endothelial growth factor signaling through VEGFR-1, VEGFR-2, and VEGFR-3 represents a principal mechanism by which tumors remodel the microenvironment to support abnormal vasculature, promote hypoxia, and impair drug delivery [4,5]. Targeting VEGFR-mediated angiogenesis has therefore emerged as an important therapeutic strategy for disrupting tumor microenvironment signaling and improving outcomes across multiple GI malignancies [4,5]. VEGFR signaling also plays key roles in normal vascular, renal, and epithelial homeostasis, pharmacologic inhibition predictably results in a characteristic spectrum of treatment-related toxicities.

Fruquintinib is a highly selective oral tyrosine kinase inhibitor targeting VEGFR-1, VEGFR-2, and VEGFR-3 that has demonstrated survival benefit in previously treated metastatic colorectal cancer in the phase III FRESCO and FRESCO-2 trials, leading to regulatory approval and widespread clinical adoption [1,2,6]. Activity beyond colorectal cancer has also been demonstrated in the FRUTIGA trial, in which fruquintinib combined with paclitaxel improved outcomes in advanced gastric and gastroesophageal junction adenocarcinoma [3]. As fruquintinib becomes increasingly incorporated into later-line and combination treatment strategies aimed at sustained angiogenesis suppression within the tumor microenvironment, clinicians face practical questions regarding which toxicities most strongly influence treatment tolerability and how these risks should be prioritized to maintain therapeutic benefit [7].

VEGFR inhibition is associated with a well-described spectrum of adverse events, including hypertension, proteinuria, hemorrhage, and dermatologic toxicity such as hand–foot skin reaction and palmar–plantar erythrodysesthesia. Although these toxicities are recognized across VEGFR-targeted therapies, their reported incidence and severity vary substantially between individual trials, tumor types, and treatment contexts [8,9]. Bleeding and venous thromboembolism represent competing clinical risks in patients with advanced GI malignancies receiving angiogenesis inhibitors, complicating decisions regarding monitoring intensity and anticoagulation. Trial-level estimates for these events remain inconsistent and are frequently underpowered due to low event counts [10].

Existing safety evaluations of VEGFR-targeted therapies often assess adverse events within single trials or focus on specific toxicity domains, limiting integrated assessment of the full toxicity profile in later-line and combination settings where multiple adverse events may occur concurrently and collectively influence clinical management [9,10]. As fruquintinib use expands following regulatory approval, pooled randomized evidence is needed to clarify the relative importance of these toxicities, quantify their magnitude, and better define the uncertainty surrounding bleeding and thrombotic outcomes in the context of tumor microenvironment–targeted angiogenesis inhibition.

The objective of this study was to synthesize randomized phase II and phase III trial data to identify the dominant grade ≥3 toxicities associated with fruquintinib and to clarify the relative risks of hemorrhage and venous thromboembolism in pooled randomized evidence, thereby informing evidence-based monitoring priorities, patient selection, and toxicity-guided management in contemporary gastrointestinal oncology practice.

## 2 Materials and Methods

### 2.1 Protocol and Reporting Standards

This study was conducted according to a prespecified analytic plan developed prior to data extraction. The protocol was not registered in PROSPERO; the analytic framework and study objectives were defined in advance and are reported in accordance with PRISMA 2020 guidelines. A completed PRISMA 2020 checklist and the detailed analytic framework are provided in the Supplement File.

### 2.2 Search Strategy

A systematic search of MEDLINE, EMBASE, and the Cochrane Central Register of Controlled Trials was performed from database inception through 1 January 2026 to identify randomized controlled trials evaluating the safety of fruquintinib in gastrointestinal malignancies. The search strategy combined controlled vocabulary and free-text terms related to fruquintinib, gastrointestinal cancer, colorectal cancer, gastric cancer, gastroesophageal junction cancer, and treatment-related toxicities, including hemorrhage, venous thromboembolism, hypertension, proteinuria, and hand–foot skin reaction or palmar–plantar erythrodysesthesia.

Conference abstracts from the American Society of Clinical Oncology and the European Society for Medical Oncology annual meetings were screened to ensure identification of all potentially eligible randomized trials. Abstract-only reports were not included in the quantitative synthesis unless a corresponding full-text publication with extractable safety data was available.

The complete electronic search strategies for all databases, including full search strings, Boolean operators, and applied limits, are provided in the Supplement File.

### 2.3 Eligibility Criteria

Eligible studies were randomized phase II or phase III clinical trials enrolling adult patients with gastrointestinal malignancies, including metastatic colorectal cancer, gastric cancer, or gastroesophageal junction adenocarcinoma, that compared fruquintinib-containing regimens with placebo or standard therapy. Studies were required to report at least one prespecified adverse event of interest, including hemorrhage, venous thromboembolism, hypertension, proteinuria, or hand–foot skin reaction or palmar–plantar erythrodysesthesia.

Non-randomized studies, single-arm trials, observational studies, case reports, and case series were excluded. Trials evaluating fruquintinib in non-gastrointestinal malignancies or lacking extractable safety data were also excluded. To minimize bias and ensure methodological consistency, the analysis was restricted to randomized phase II or phase III superiority trials with placebo or standard-of-care control arms. Post-approval real-world datasets and expanded-access programs were excluded because these study designs may introduce additional heterogeneity and confounding relative to randomized controlled trials.

### 2.4 Study Selection

**Figure 1:**
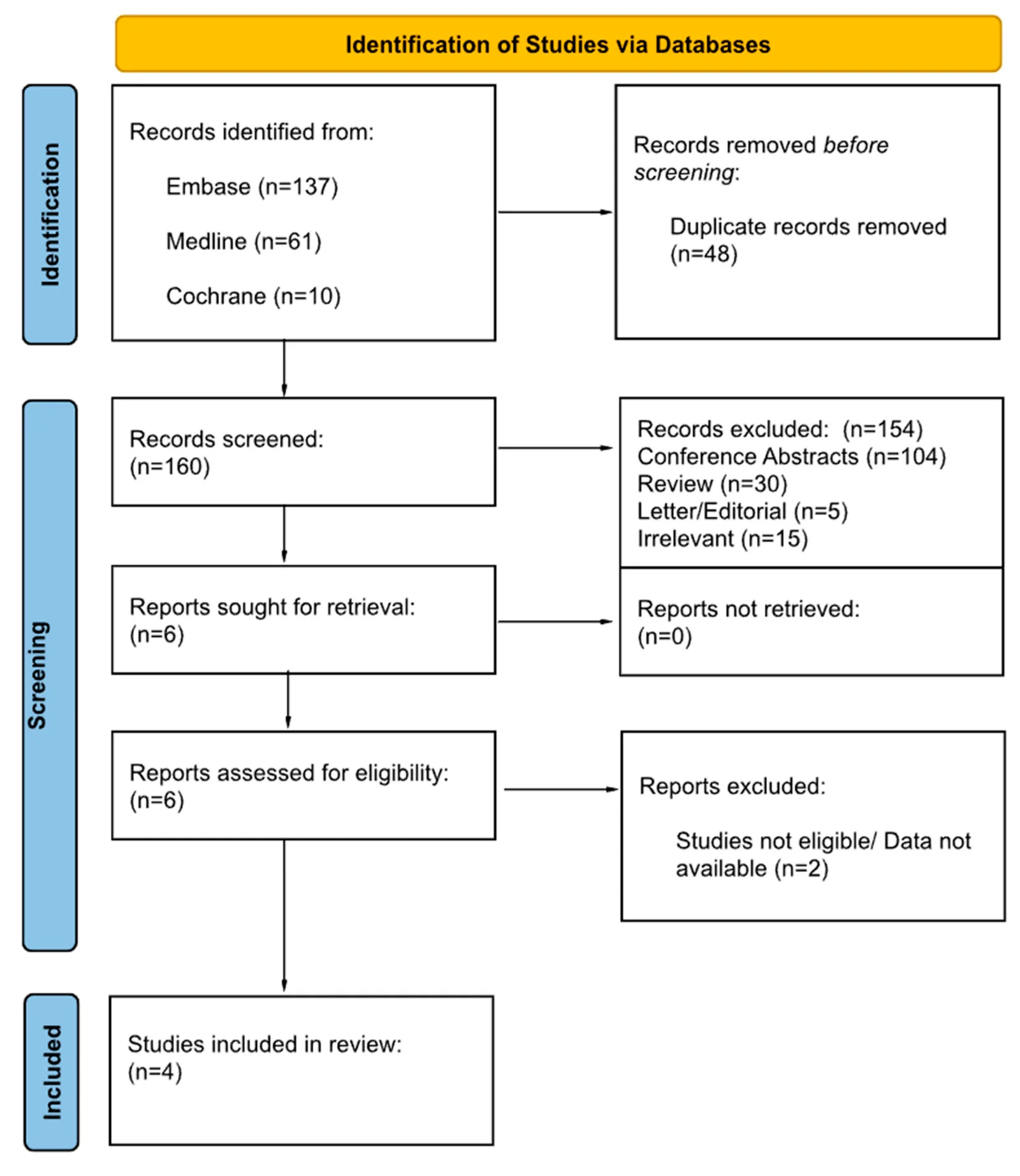
PRISMA flow diagram of study identification, screening, and inclusion.

Study selection was conducted in two stages. Titles and abstracts were first screened to identify potentially eligible studies. Full-text review was then performed to confirm eligibility based on the predefined inclusion criteria. Disagreements at either stage were resolved through consensus discussion. The study selection process is summarized in a PRISMA flow diagram (Fig. 1).

### 2.5 Data Extraction

Two investigators independently extracted data using a standardized data collection form. Extracted variables included trial design, study phase, geographic region, sample size, cancer type, treatment regimens, dosing schedules, and duration of follow-up. Safety outcomes were extracted using the trial-reported safety population as the denominator, with events recorded as the number of adverse events and the total number of patients in each treatment arm for each toxicity category. Outcomes were stratified by any-grade and grade ≥3 events when reported.

Additional variables extracted, when available, included relative dose intensity, frequency of dose reductions or interruptions, and treatment discontinuation due to adverse events. Any discrepancies between reviewers were resolved through discussion and consensus.

### 2.6 Risk of Bias Assessment

Risk of bias was assessed at the study level using the Cochrane Risk of Bias tool (RoB 2) for randomized trials. Domains evaluated included the randomization process, deviations from intended interventions, missing outcome data, measurement of outcomes, and selection of reported results. Each domain was classified as low risk of bias, some concerns, or high risk of bias according to RoB 2 guidance.

### 2.7 Statistical Analysis

For each adverse event, pooled risk ratios (RRs) with 95% confidence intervals (CIs) were calculated using Mantel–Haenszel random-effects models to account for potential between-study heterogeneity. Between-study variance (τ^2^) was estimated using a restricted maximum likelihood estimator. Statistical heterogeneity was assessed using the I^2^ statistic, with values of approximately 25%, 50%, and 75% representing low, moderate, and high heterogeneity, respectively.

For outcomes containing zero-event cells, a continuity correction of 0.5 was applied at the study-arm level. Given the low incidence of venous thromboembolism and several grade ≥3 adverse events, sensitivity analyses using alternative approaches for sparse data were prespecified, including Peto odds ratios. Because only a small number of trials were eligible, Hartung–Knapp adjustment was not applied. For rare-event outcomes, interpretation emphasized confidence interval width, absolute event rates, and consistency across sensitivity analyses rather than point estimates alone.

Prespecified sensitivity analyses included leave-one-out analyses and exclusion of the FRUTIGA trial to assess the potential impact of combination therapy with paclitaxel on pooled estimates. Exploratory stratified analyses were performed where trial-level data permitted, including analyses within metastatic colorectal cancer cohorts and descriptive comparisons between fruquintinib monotherapy trials and the FRUTIGA combination-therapy trial.

All analyses were conducted using R software (version 4.3.2). A two-sided alpha level of 0.05 was used for statistical significance. Assessment of publication bias was not performed because fewer than ten studies were included.

Because the FRUTIGA trial evaluated fruquintinib in combination with paclitaxel, attribution of adverse events to individual agents cannot be determined directly from trial-level safety data. The randomized design compares fruquintinib plus paclitaxel versus paclitaxel alone, allowing estimation of the incremental toxicity associated with the addition of fruquintinib. Sensitivity analyses excluding FRUTIGA were therefore performed to evaluate whether the inclusion of combination therapy materially influenced pooled estimates.

## 3 Results

### 3.1 Trial Characteristics

Four randomized controlled trials met the inclusion criteria, comprising 1872 patients, of whom 1137 received fruquintinib. Three trials were conducted in China, and one was a global phase III study enrolling patients across North America, Europe, Japan, and Australia. Most studies evaluated fruquintinib monotherapy in metastatic colorectal cancer, whereas the FRUTIGA trial evaluated fruquintinib in combination with paclitaxel in patients with gastric or gastroesophageal junction adenocarcinoma. Key characteristics of the included trials are summarized in Table 1.

**Table 1:** Characteristics of randomized phase II and phase III trials evaluating fruquintinib in gastrointestinal malignancies.

Study	Design	Patients (Fruquintinib/Control)	Cancer Type	Treatment Regimen	Primary Outcome
FRESCO [1]	Phase III, randomized, double-blind, placebo-controlled	278/138	Metastatic colorectal cancer (mCRC)	Fruquintinib 5 mg oral daily, 3 weeks on/1 week off, +best supportive care (BSC)	Overall Survival (OS)
FRESCO-2 [2]	Phase III, international, randomized, double-blind	461/230	Refractory metastatic colorectal cancer (mCRC)	Fruquintinib 5 mg PO daily, 3 weeks on/1 week off, +BSC	Overall Survival (OS)
FRUTIGA [3]	Phase III, randomized, double-blind	351/352	Gastric or gastroesophageal junction adenocarcinoma (G/GEJ)	Fruquintinib 4 mg PO daily, 3 weeks on/1 week off, +paclitaxel 80 mg/m^2^ IV on days 1, 8, 15 per cycle	Progression-Free Survival (PFS)
Xu et al. (2017) [11]	Phase II, randomized, double-blind	47/24	Metastatic colorectal cancer (mCRC)	Fruquintinib 5 mg PO daily, 3 weeks on/1 week off, +BSC	Progression-Free Survival (PFS)

### 3.2 Baseline Patient Characteristics

Baseline demographic characteristics, including age and Eastern Cooperative Oncology Group (ECOG) performance status, were broadly similar across the included trials. Detailed baseline vascular risk factors, anticoagulant exposure, and bleeding-risk variables were not consistently reported in the published datasets and therefore could not be systematically compared across studies.

Across trials, the median patient age ranged from the early to mid-sixties, and most participants had ECOG performance status 0–1. All trials enrolled heavily pretreated populations, with common prior exposure to anti–vascular endothelial growth factor therapies. Baseline hypertension and renal dysfunction were uncommon. By disease context, patients with gastric or gastroesophageal junction adenocarcinoma may have greater baseline mucosal bleeding vulnerability than metastatic colorectal cancer cohorts, although these risk factors were not systematically reported across studies.

### 3.3 Dose Intensity and Treatment Modifications

Reporting of dose intensity, dose reductions, and treatment discontinuation due to adverse events was inconsistent across trials, precluding quantitative pooling. Available trial-level data suggest that hypertension, proteinuria, and dermatologic toxicity were among the most frequent reasons for treatment modification. Pooled estimates of relative dose intensity and treatment discontinuation rates were not consistently reported across studies. In the FRUTIGA trial, higher rates of vascular and renal adverse events were observed relative to the monotherapy trials, although attribution to fruquintinib versus paclitaxel cannot be determined from trial-level safety data.

### 3.4 Clinically Actionable Grade 3 or Higher Toxicities

#### 3.4.1 Hemorrhage

Fruquintinib was associated with a numerically higher incidence of grade ≥3 hemorrhage in the overall pooled cohort. Grade ≥3 hemorrhage occurred in 2.30% (26/1131) of patients receiving fruquintinib compared with 1.35% (10/741) of control patients. The pooled risk ratio for grade ≥3 hemorrhage was 1.82 (95% CI 0.92–3.61; *p* = 0.09), indicating a numerical increase that did not reach statistical significance.

In the metastatic colorectal cancer subgroup, the pooled risk ratio for grade ≥3 hemorrhage was 1.06 (95% CI 0.35–3.24; *p* = 0.91), with similarly low absolute event rates. These findings indicate that although hemorrhagic events occurred during VEGFR inhibition, absolute rates were low and the pooled estimate did not demonstrate a statistically significant increase in grade ≥3 hemorrhage across the randomized trials.

**Figure 2:**
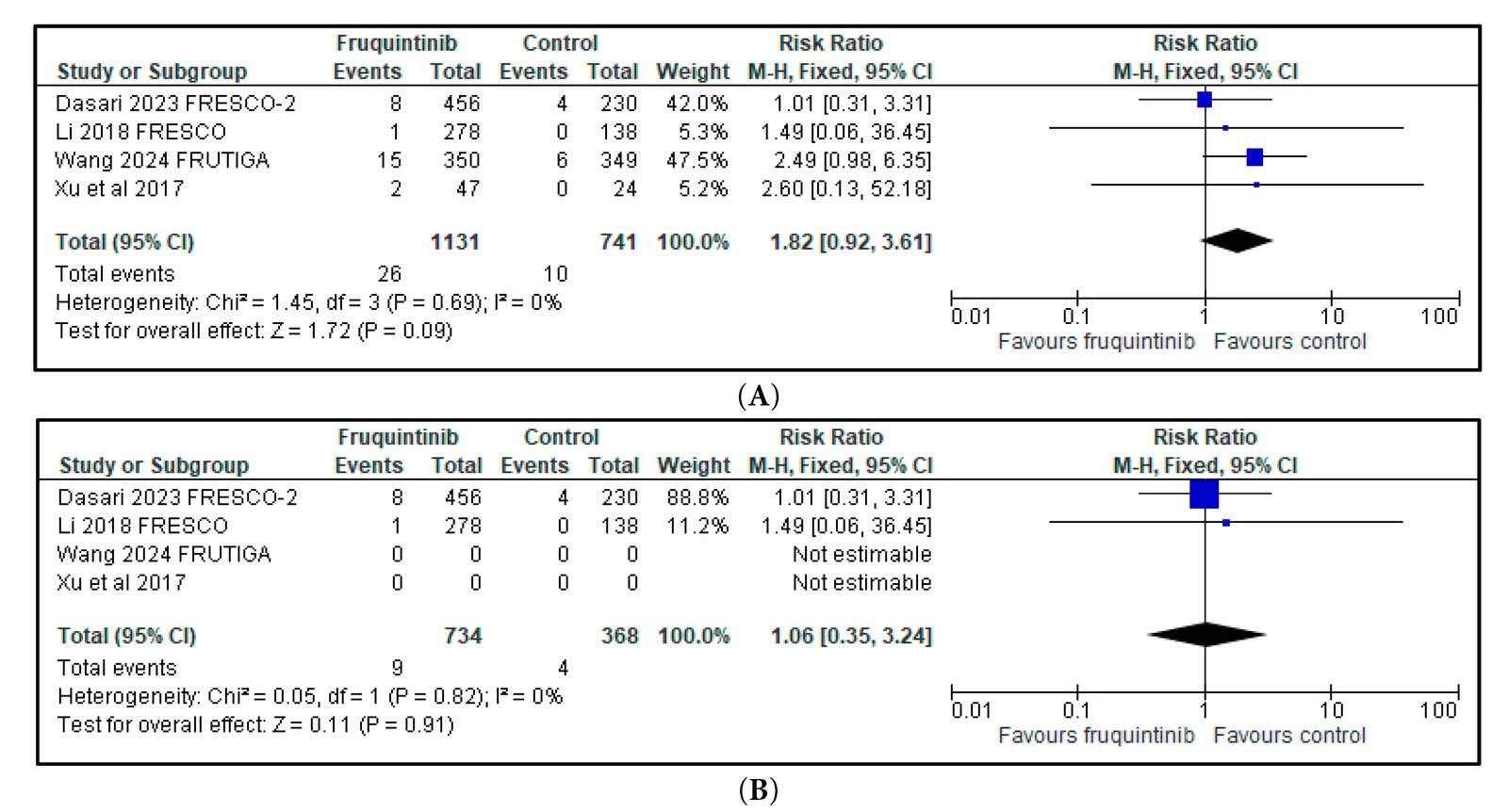
Forest plots of hemorrhage with fruquintinib versus control. (**A**) Overall grade ≥3 hemorrhage in GI cancers, and (**B**) metastatic colorectal cancer subgroup grade ≥3 hemorrhage [1,2,3,11].

Active bleeding was an exclusion criterion in the included studies; nevertheless, hemorrhagic events were observed during treatment. Between-study heterogeneity for hemorrhage outcomes was low to moderate, as shown in Fig. 2 [1,2,3,11].

#### 3.4.2 Hypertension

The incidence of any-grade HTN was higher in the fruquintinib group, 34.92% vs. 7.55% (RR 3.96; 95% CI: 3.05–5.13; *p* < 0.00001). The rate of high-grade HTN was 13.96% vs. 1.21% (RR 9.01; 95% CI: 4.67–17.40; *p* < 0.00001).

In the metastatic colorectal cancer subgroup, incidence of any-grade HTN was 43.02% vs. 10.45% (RR 3.97; 95% CI: 2.95–5.33; *p* < 0.00001). The incidence of high-grade HTN in colorectal patients was 17.28% vs. 1.27% (RR 12.06; 95% CI: 5.19–28.01; *p* < 0.00001).

**Figure 3:**
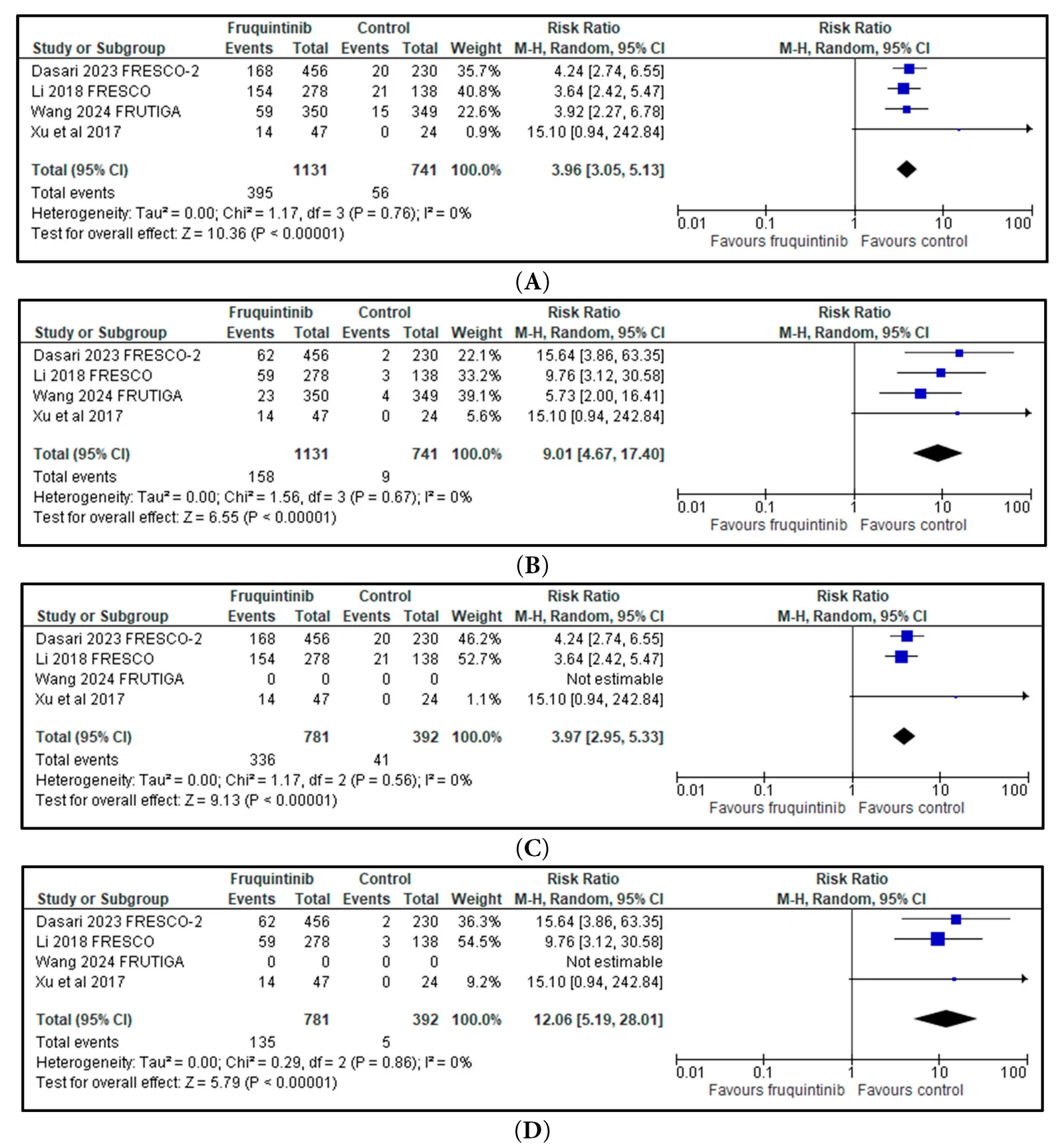
Forest plots of hypertension with fruquintinib versus control. (**A**) Any grade hypertension in GI cancers, (**B**) high grade hypertension in GI cancers, (**C**) any-grade hypertension in colorectal cancer, and (**D**) high grade hypertension in colorectal cancer subgroup [1,2,3,11].

Between-study heterogeneity was low across hypertension analyses (I^2^ = 0%), indicating consistent treatment effects across trials (Fig. 3) [1,2,3,11].

#### 3.4.3 Hand-Foot Skin Reaction and Palmar Plantar Erythrodysesthesia

The incidence of any-grade HFSR or PPE in GI cancers was higher in the fruquintinib group compared to the placebo group, 27.93% (316/1131) vs. 3.64% (27/741) (RR, 7.64; 95% CI: 4.08–14.33; *p* < 0.00001). In the overall pooled cohort, grade ≥3 hand–foot skin reaction or palmar–plantar erythrodysesthesia (HFSR/PPE) occurred in 8.50% (97/1131) of patients receiving fruquintinib and 0% (0/741) of control patients, corresponding to a pooled risk ratio of 26.00 (95% CI 6.42–105.29; *p* < 0.00001). This large relative risk estimate reflects the absence of events in several control arms, and therefore should be interpreted alongside the absolute event incidence observed in the included trials.

**Figure 4:**
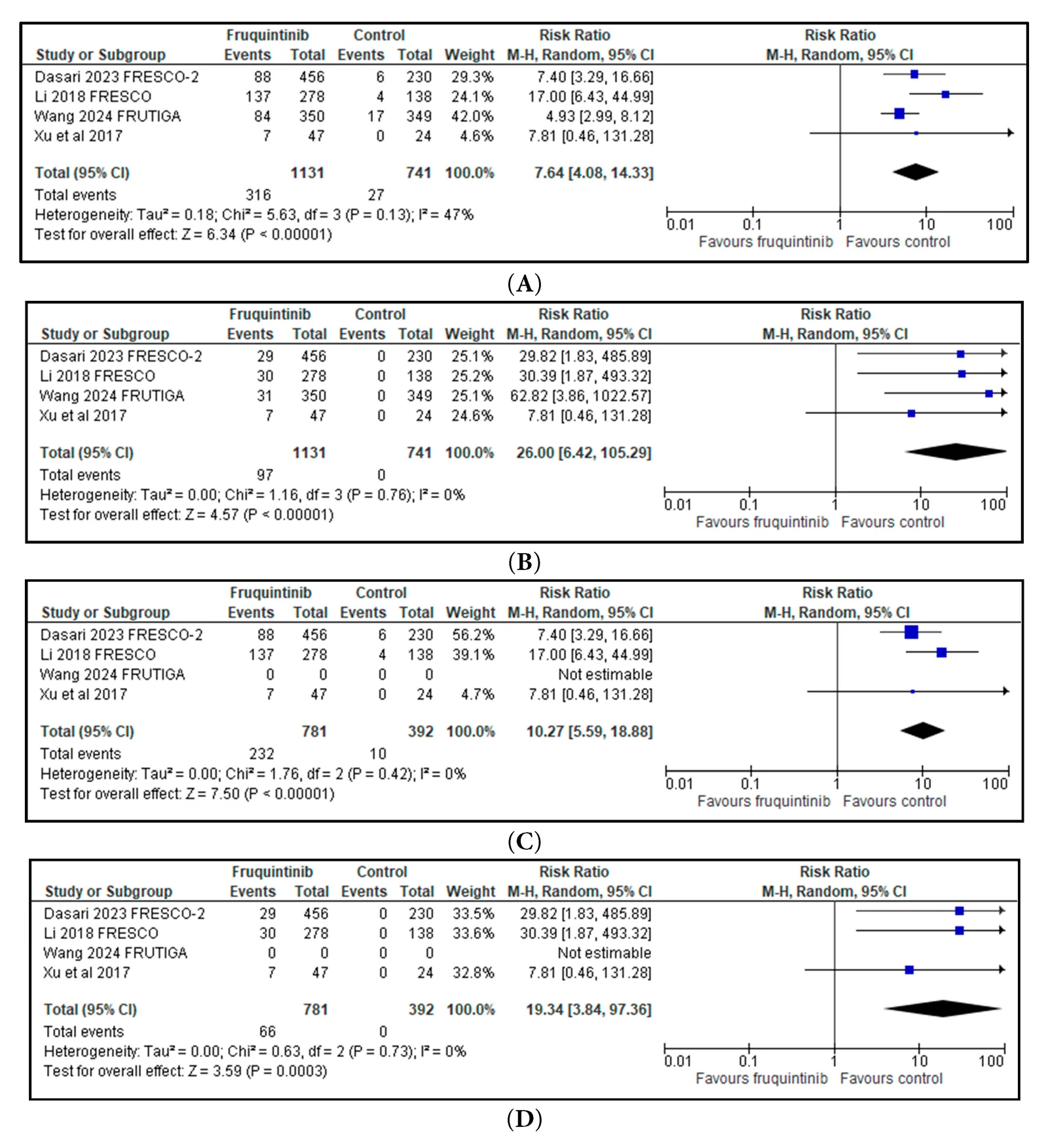
Forest plots of hand–foot skin reaction or palmar–plantar erythrodysesthesia (HFSR/PPE) with fruquintinib versus control. (**A**) Any-grade HFSR/PPE in GI cancers, (**B**) high-grade HFSR/PPE in GI cancers, (**C**) any-grade HFSR/PPE in colorectal cancers, and (**D**) high-grade HFSR/PPE in colorectal cancers [1,2,3,11].

In the mCRC subgroup analysis, the higher incidence of any-grade of HFSR or PPE in the fruquintinib arm was also statistically significant, 29.70% (232/781) vs. 2.55% (10/392) (RR, 10.27; 95% CI: 5.59–18.88; *p* < 0.00001). Grade ≥3 HFSR/PPE occurred in 8.45% of patients receiving fruquintinib and 0% of control patients, corresponding to a pooled risk ratio of 19.34 (95% CI 3.84–97.36; *p* = 0.0003). Between-study heterogeneity was minimal across analyses (I^2^ = 0%), indicating consistent dermatologic toxicity signals across trials (Fig. 4) [1,2,3,11].

### 3.5 Proteinuria

Any-grade proteinuria was also increased with fruquintinib. In the overall pooled cohort, any-grade proteinuria in GI cancers occurred in 27.30% of patients receiving fruquintinib compared with 16.19% of control patients, corresponding to a pooled risk ratio of 1.89 (95% CI 1.30–2.74; *p* = 0.0008). In GI cancer group, high-grade proteinuria occurred 1.85% vs. 0.53% (RR, 2.49; 95% CI: 0.86–7.16; *p* = 0.09).

In the metastatic colorectal cancer subgroup, the corresponding any-grade proteinuria rates were 25.22% and 11.73%, with a pooled risk ratio of 2.29 (95% CI 1.17–4.51; *p* = 0.02). In the colorectal subgroup, high-grade proteinuria occured 2.17% vs. 0.51% (RR, 2.87; 95% CI: 0.74–11.12; *p* = 0.13). 

**Figure 5:**
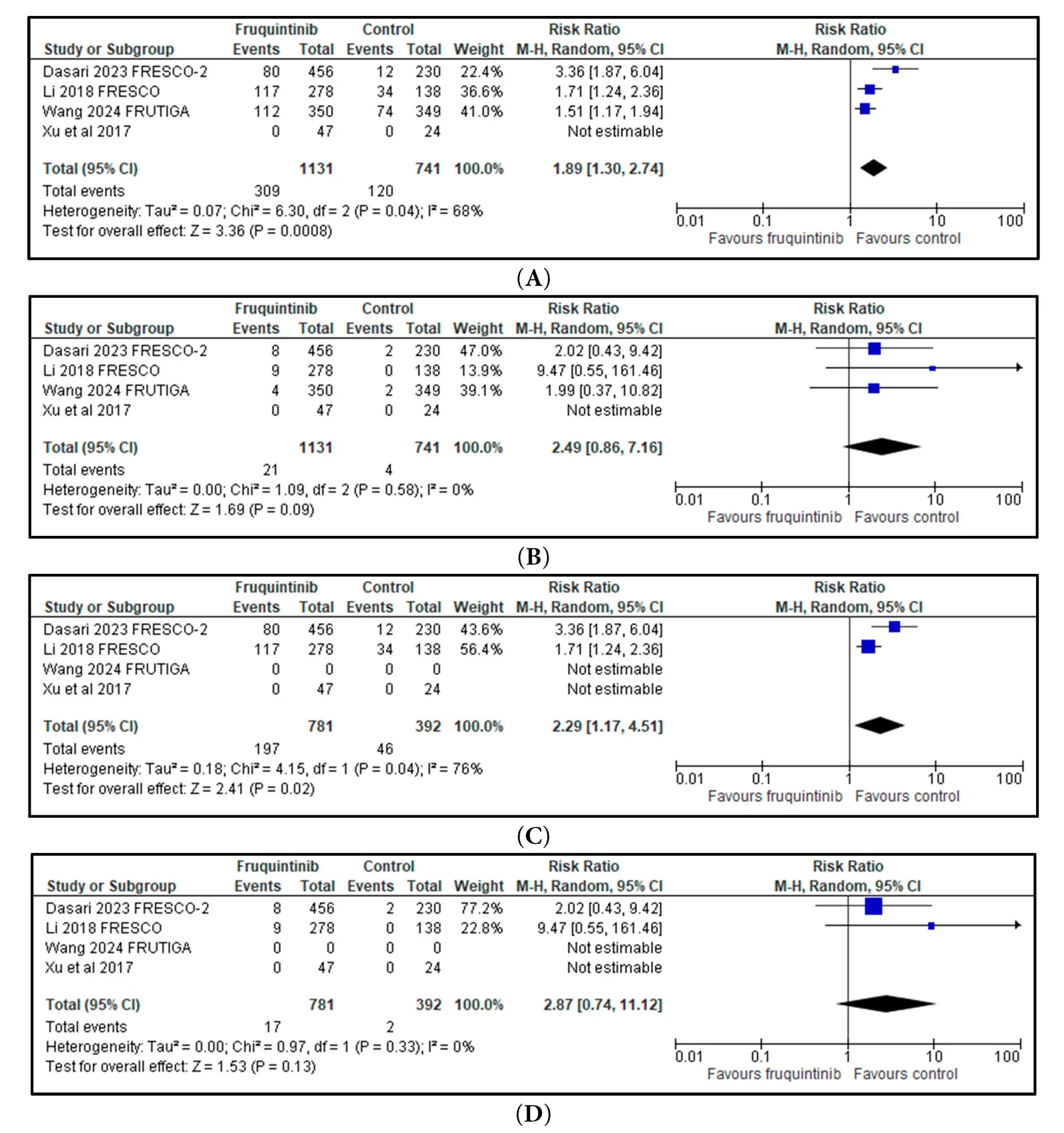
Forest plots of proteinuria with fruquintinib versus control. (**A**) Overall any-grade proteinuria in GI cancers, (**B**) high-grade proteinuria in GI cancers, (**C**) overall any-grade proteinuria in colorectal cancer, and (**D**) high-grade proteinuria in colorectal cancer [1,2,3,11].

Grade ≥3 proteinuria was uncommon across trials and did not differ significantly between treatment groups. Confidence intervals were wide because of the low number of events, reflecting the limited precision of pooled estimates for this endpoint (Fig. 5) [1,2,3,11].

### 3.6 Venous Thromboembolism (VTE)

Venous thromboembolism (VTE) events were infrequent across the included trials. In the overall pooled cohort, any-grade VTE occurred in 2.74% of patients receiving fruquintinib compared with 2.15% of control patients, corresponding to a pooled risk ratio of 1.23 (95% CI 0.53–2.88; *p* = 0.63). Grade ≥3 VTE in GI cancers occurred in 1.67% of patients receiving fruquintinib and 0.67% of control patients, yielding a pooled risk ratio of 1.96 (95% CI 0.52–7.36; *p* = 0.32).

Incidence of any-grade VTE events in colorectal was 2.68% vs. 1.53% (RR, 0.95; 95% CI: 0.09–9.67; *p* = 0.97). Incidence of high-grade VTE events in colorectal subgroup was 1.79% vs. 0.76% (RR, 1.08; 95% CI: 0.06–19.98; *p* = 0.96).

**Figure 6:**
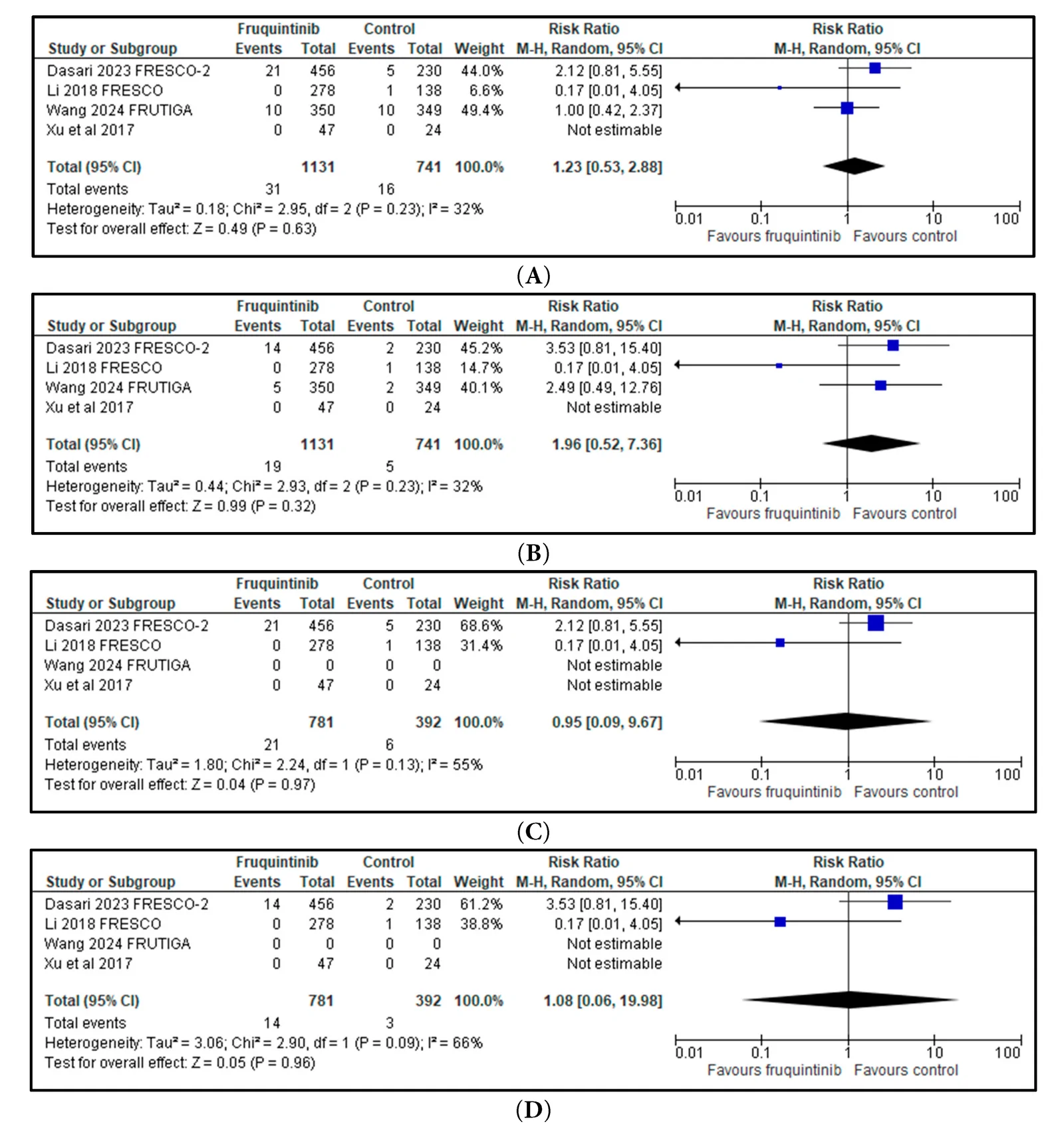
Forest plots of venous thromboembolism (VTE) with fruquintinib versus control. (**A**) Overall any-grade VTE in GI cancers, (**B**) overall high-grade VTE in GI cancers, (**C**) overall any-grade VTE in colorectal cancers, and (**D**) overall high-grade VTE in colorectal cancers [1,2,3,11].

Across VTE analyses, confidence intervals were wide because of the low number of observed events, indicating limited statistical precision and substantial uncertainty in the pooled estimates, particularly in subgroup analyses (Fig. 6) [1,2,3,11].

### 3.7 Sensitivity and Subgroup Analyses

Sensitivity analyses, including leave-one-out analyses and exclusion of the FRUTIGA trial, did not materially alter the direction of the primary safety signals for grade ≥3 hypertension and dermatologic toxicity. For rare-event outcomes such as hemorrhage and VTE, effect estimates remained imprecise across sensitivity analyses, and no analysis demonstrated a statistically significant increase. Exploratory stratified analyses by cancer type and treatment regimen are summarized in Fig. 2, Fig. 3, Fig. 4, Fig. 5 and Fig. 6.

## 4 Discussion

### 4.1 Principal Findings

This pooled analysis of randomized phase II and III trials characterizes a consistent toxicity profile for fruquintinib across gastrointestinal malignancies that reflects the biologic effects of VEGFR inhibition [1,2,3,11]. Hypertension and dermatologic toxicity represent the dominant grade ≥3 adverse events, with large effect sizes and uniform findings across studies, defining a vascular and microvascular pattern that is predictable in onset and directly relevant to treatment management [2,4]. These findings are consistent with a pharmacodynamic response to sustained angiogenesis inhibition, in which endothelial signaling disruption drives systemic vascular effects [9,12]. Renal toxicity presents primarily as low-grade proteinuria, indicating a parallel manifestation of endothelial injury that is generally less likely to limit treatment exposure [4,9]. Bleeding and thrombotic events remain infrequent within the available randomized data, and the wide confidence intervals surrounding these estimates reflect limited event counts rather than a clearly defined risk signal [2,4]. The overall pattern supports a toxicity profile dominated by mechanism-based adverse events that can be anticipated and managed, while vascular complications beyond these core effects remain less clearly defined.

### 4.2 Clinical Significance of Hemorrhagic Risk During VEGFR Inhibition

Hemorrhagic events occurred at low absolute rates in this pooled analysis despite sustained VEGFR inhibition, and the numerically higher incidence observed with fruquintinib did not reach statistical significance, supporting a modest and incompletely defined signal rather than a clear safety liability [2,4]. This pattern is biologically plausible, as VEGF signaling is central to endothelial homeostasis and vascular repair, and its inhibition disrupts mucosal microvascular integrity through reduced nitric oxide bioavailability and increased capillary fragility [9,12]. Interpretation of these findings is influenced by trial design, since patients with active or clinically significant bleeding were excluded at enrollment, likely attenuating observed event rates and limiting applicability to higher-risk populations [1,2]. Attribution remains complex in gastrointestinal malignancies, where baseline bleeding risk is elevated due to tumor-associated mucosal disruption, luminal invasion, and cumulative vascular injury from prior therapies [3]. Evidence across VEGFR-targeted therapies demonstrates a similar profile, with hemorrhage recognized as a class effect while severe and fatal events remain uncommon in randomized settings [9,12]. These findings support an approach centered on individualized risk assessment and monitoring rather than routine avoidance of therapy, with particular attention to patients with recent bleeding, mucosal vulnerability, or concurrent anticoagulant use [4,12].

### 4.3 Hypertension as the Dominant On-Target Toxicity of Fruquintinib

Hypertension represents the most consistent and reproducible toxicity observed in this analysis, supported by large effect sizes and minimal heterogeneity across trials, indicating a true class effect of VEGFR inhibition rather than study-specific variation [1,2]. This pattern is mechanistically well established, as VEGF pathway blockade reduces endothelial nitric oxide production, leading to impaired vasodilation, increased systemic vascular resistance, and structural microvascular changes [9,12]. The magnitude of risk observed here is concordant with findings from the FRESCO and FRESCO-2 trials, where hypertension was among the most frequent high-grade adverse events and a key determinant of treatment modification [1,2]. In clinical settings, hypertension often develops early after treatment initiation and directly contributes to dose interruption and reduction, with implications for maintaining therapeutic exposure [4]. The consistency, timing, and biologic basis of this toxicity support its classification as an on-target pharmacodynamic effect of angiogenesis inhibition rather than a nonspecific adverse event. These findings highlight the importance of baseline cardiovascular assessment, optimization of blood pressure prior to treatment, and structured early monitoring to facilitate timely intervention and minimize disruption of therapy [4,12].

### 4.4 Impact of HFSR/PPE on Treatment Adherence and Quality of Life

Dermatologic toxicity, specifically hand–foot skin reaction or palmar–plantar erythrodysesthesia (HFSR/PPE), represents a prominent adverse effect associated with fruquintinib, characterized by high relative risk estimates that require interpretation in the context of moderate absolute event rates. In the FRESCO and FRESCO-2 trials, grade ≥3 HFSR/PPE occurred in approximately 11% and 6% of treated patients, respectively, supporting its clinical relevance while contextualizing the magnitude of risk [1,2]. The underlying mechanism reflects VEGFR inhibition–mediated microvascular injury, in which capillary endothelial damage in areas exposed to repetitive pressure and friction results in impaired perfusion, keratinocyte dysfunction, and the characteristic inflammatory skin changes observed in this syndrome [9,13]. This toxicity pattern is well established across VEGFR-targeted therapies and reflects a consistent, mechanism-based class effect rather than an idiosyncratic drug-specific reaction [1,2]. Although rarely associated with life-threatening complications, HFSR/PPE exerts a disproportionate impact on patient quality of life through pain, impaired mobility, and reduced functional capacity, and is a leading cause of dose interruption and reduction in clinical practice [4]. These effects place HFSR/PPE among the most important determinants of treatment adherence and continuity. Management requires a proactive approach centered on early symptom recognition, structured dermatologic monitoring during initial treatment cycles, and timely implementation of supportive care measures, including emollient-based skin protection, topical therapies, and protocol-guided dose modification to maintain therapeutic exposure [4,13].

### 4.5 Renal Manifestations of VEGFR Blockade

Proteinuria was frequently observed in this analysis, driven primarily by increases in any-grade events, while grade ≥3 toxicity remained uncommon, supporting its classification as a predictable and largely monitorable manifestation of VEGFR inhibition rather than a major treatment-limiting complication [1,2]. The underlying mechanism reflects injury to glomerular endothelial and podocyte compartments, where VEGF signaling is essential for maintaining filtration barrier integrity; its inhibition disrupts this homeostasis and increases permeability to circulating proteins in a manner related to treatment exposure [9,14]. This process shares a common biologic basis with VEGFR inhibitor–associated hypertension, with both toxicities arising from systemic endothelial dysfunction and impaired nitric oxide–mediated vascular regulation [12]. Similar patterns have been consistently reported across VEGFR-targeted therapies, supporting proteinuria as a pharmacologic class effect rather than a drug-specific phenomenon [1,9]. In clinical practice, proteinuria is typically manageable and rarely necessitates treatment discontinuation, with intervention generally reserved for higher-grade or progressive renal toxicity [4]. These findings support incorporation of baseline urinalysis with quantitative protein assessment, followed by longitudinal monitoring throughout treatment to enable early detection of renal injury and guide dose adjustment when clinically indicated [4,12].

### 4.6 Interpreting Thrombotic Risk in Fruquintinib-Treated Patients

Venous thromboembolism events were infrequent in this analysis, and pooled risk estimates did not reach statistical significance; the low number of observed events and wide confidence intervals limit statistical power and do not exclude a clinically meaningful thrombotic effect of fruquintinib [2,4]. Interpretation is constrained by imprecision inherent to rare-event outcomes, and failure to detect a significant association in this setting reflects limited precision rather than established safety [15]. The biologic context remains complex, with malignancy-associated hypercoagulability coexisting alongside VEGFR inhibition–related endothelial disruption, both of which influence thrombosis through alterations in vascular integrity and endogenous anticoagulant signaling [9,12]. These processes may act in parallel, creating a setting in which thrombotic risk is shaped by both disease-related and treatment-related factors. Generalizability is limited by the predominance of East Asian cohorts in the included trials, where the baseline prevalence of inherited thrombophilic variants is lower than in Western populations, potentially contributing to lower observed event rates [1,3]. These limitations do not support an assumption of minimal thrombotic risk. Clinical management should incorporate structured VTE risk assessment, with individualized decisions regarding thromboprophylaxis that balance the competing risks of thrombosis and bleeding in the context of patient-specific characteristics and disease burden [12,16].

### 4.7 Integrated Toxicity Profile of Fruquintinib

The toxicity profile observed with fruquintinib reflects a coherent phenotype of VEGFR inhibition rather than a collection of unrelated adverse events, with individual toxicities mapping onto interconnected vascular axes. Hypertension and proteinuria align along a shared vascular–renal axis driven by systemic endothelial dysfunction, in which suppression of VEGF-dependent nitric oxide signaling increases vascular tone while parallel disruption within the glomerular microvasculature compromises filtration barrier integrity, producing coordinated cardiovascular and renal manifestations of a common endothelial insult [9,12]. Dermatologic toxicity, particularly HFSR/PPE, represents a complementary microvascular–dermatologic axis, arising from capillary injury in regions exposed to repetitive pressure and shear forces, where impaired vascular maintenance leads to localized ischemia, keratinocyte dysfunction, and characteristic inflammatory skin changes [13]. The co-occurrence and temporal clustering of these toxicities in clinical practice support a shared biologic origin linked to sustained angiogenesis inhibition, indicating that the safety profile of fruquintinib is best understood as a unified syndrome of endothelial injury with variable organ-level expression. Hemorrhagic and thrombotic events occupy a less clearly defined position within this framework, as low absolute event rates and imprecise estimates limit definitive mechanistic attribution, though the underlying endothelial disruption provides biologic continuity. Recognition of these relationships supports a systems-based approach to toxicity monitoring, integrating cardiovascular, renal, and dermatologic assessment rather than treating adverse events in isolation [4,12]. The interpretation of these toxicities as on-target pharmacodynamic effects also raises the possibility that early manifestations such as hypertension or proteinuria may reflect biologic drug activity, an observation described across antiangiogenic therapies that warrants prospective evaluation in fruquintinib-treated populations [9,17].

### 4.8 Comparative Safety Context

The safety profile of fruquintinib is best interpreted alongside other later-line therapies in metastatic colorectal cancer, where differences in target selectivity define distinct toxicity patterns. Regorafenib, a multikinase inhibitor with activity across VEGFR, RAS/RAF, PDGFR, FGFR, and KIT pathways, is associated with a broader and less selective adverse event spectrum; in the CORRECT trial, grade ≥3 toxicities included hand–foot skin reaction (17%), fatigue (15%), diarrhea (8%), and hypertension (8%), with dose modifications required in the majority of patients and hepatotoxicity emerging as an additional class-specific concern [18]. Comparative data indicate that fruquintinib is associated with a lower overall burden of high-grade treatment-related adverse events relative to regorafenib, with toxicity concentrated in a narrower set of VEGFR-mediated effects, particularly hypertension, consistent with its higher target selectivity. Trifluridine/tipiracil demonstrates a distinct toxicity profile driven by cytotoxic mechanisms, with grade ≥3 neutropenia (38%), anemia (18%), and thrombocytopenia (5%) predominating and minimal vascular or dermatologic toxicity observed [19]. The addition of bevacizumab reintroduces VEGF pathway–related toxicities, with SUNLIGHT demonstrating increased rates of hypertension alongside further amplification of myelosuppression [20]. These comparisons indicate that fruquintinib does not reduce toxicity in absolute terms but redistributes it into a more selective, mechanism-defined profile characterized by predictable endothelial effects that can be monitored and managed, distinguishing it from the broader off-target toxicities of multikinase inhibition and the hematologic burden of cytotoxic therapies [2,4].

### 4.9 Clinical Implications

Effective toxicity management is essential to preserve therapeutic exposure in later-line disease, where treatment options are limited and the margin between clinical benefit and discontinuation is narrow. Pre-treatment evaluation should focus on the dominant toxicity domains identified in this analysis. Blood pressure should be controlled before starting therapy. Contemporary guidance supports initiation of antihypertensive treatment, preferably with an angiotensin-converting enzyme inhibitor, angiotensin receptor blocker, or dihydropyridine calcium channel blocker, with a target below 130/80 mmHg during treatment [21,22]. Baseline renal assessment with urinalysis and quantitative protein measurement is recommended because proteinuria is common and often develops early [2]. Bleeding risk assessment should include tumor location, mucosal integrity, and concomitant anticoagulation, since higher-grade hemorrhagic events require treatment interruption or discontinuation [2].

Early monitoring during the first treatment cycles is critical because hypertension and dermatologic toxicity frequently appear within the initial cycle. Blood pressure should be checked at least every two weeks during early therapy, with home monitoring initiated at treatment start [22]. Proactive dermatologic care that includes patient education, routine moisturization, pressure avoidance, and early use of urea-based creams can reduce the severity of HFSR and limit the need for dose modification [13]. Maintenance of dose intensity remains a central objective because treatment benefit is closely tied to cumulative exposure. In FRESCO-2, dose interruptions and reductions were common, yet high relative dose intensity was maintained with structured toxicity management [2,4].

Risk-adapted decision making is required when addressing competing vascular complications. Thrombotic risk should be assessed using validated tools such as the Khorana score, with individualized decisions regarding thromboprophylaxis that account for concurrent bleeding risk, particularly in gastrointestinal malignancies [16]. Selection of anticoagulation should consider the higher bleeding risk observed with direct oral anticoagulants in this population [12]. This integrated approach, based on pre-treatment optimization, early structured monitoring, and calibrated dose adjustment, supports sustained fruquintinib exposure while maintaining acceptable tolerability.

### 4.10 Methodological Considerations

This analysis carries several methodological limitations that should inform interpretation of its findings. The use of aggregated trial-level data rather than individual patient data limits adjustment for baseline comorbidities, concomitant therapies, and time-to-event variation, and restricts evaluation of patient-level interactions that may influence toxicity risk [22]. Variability in trial design, eligibility criteria, and adverse event reporting further contributes to heterogeneity that cannot be fully addressed within an aggregated framework.

Inclusion of the FRUTIGA trial introduces additional complexity. Fruquintinib was administered in combination with paclitaxel, and attribution of specific toxicities to the individual agents cannot be determined. Paclitaxel has independent hematologic, neurologic, and vascular effects that may influence observed adverse event rates, particularly for endpoints such as neutropenia, hemorrhage, and peripheral neuropathy [3].

Interpretation of rare-event outcomes is limited by sparse data. Hemorrhage and venous thromboembolism occurred infrequently across trials, resulting in wide confidence intervals and reduced statistical precision. These estimates should be considered indeterminate rather than negative, as standard meta-analytic methods are challenged by low event counts and may yield unstable results in this setting [15].

Generalizability is influenced by the geographic composition of the included studies. The evidence base is weighted toward East Asian populations, with FRESCO and FRUTIGA conducted in Chinese cohorts and FRESCO-2 including a substantial proportion of Asian patients [2,4]. Baseline thrombotic risk differs across populations, with lower prevalence of inherited thrombophilias such as Factor V Leiden and prothrombin G20210A mutations in East Asian populations compared with Western cohorts, contributing to reduced background rates of venous thromboembolism [23,24]. These differences may attenuate observed event rates and limit extrapolation of thrombotic risk to broader clinical settings. This heterogeneity in reporting standards and eligibility criteria may introduce bias and limits the generalizability of pooled toxicity estimates.

### 4.11 Future Directions

Further work should extend beyond trial-level synthesis to approaches that allow more precise characterization of fruquintinib-associated toxicity and its clinical modifiers. Individual participant data meta-analysis would enable adjustment for baseline comorbidities, prior therapies, and time-dependent drug exposure, and would allow evaluation of patient-level interactions that cannot be assessed using aggregate data. This approach is particularly relevant for rare outcomes such as hemorrhage and venous thromboembolism, where current estimates remain limited by sparse events and imprecision [25,26,27].

Real-world validation is also essential. Clinical trial populations exclude patients with uncontrolled hypertension, active bleeding, recent thromboembolism, and significant organ dysfunction. These groups are often at highest risk for VEGFR inhibitor–associated toxicities. Registry-based and observational studies can define toxicity incidence under routine clinical conditions, including the influence of comorbidity burden, polypharmacy, and variation in monitoring and dose modification practices [2,4].

Biomarker-driven toxicity prediction represents a key translational opportunity. Hypertension induced by VEGFR inhibition has been associated with improved outcomes in other antiangiogenic therapies, and proteinuria may reflect glomerular endothelial engagement. Early changes in blood pressure or urinary protein excretion may therefore serve as indicators of biologic drug activity or predictors of toxicity risk. Prospective studies are needed to determine whether these signals can guide dose titration or identify patients most likely to benefit from treatment [17,21].

Safety evaluation in combination regimens requires further study. Fruquintinib is increasingly being combined with cytotoxic chemotherapy, immune checkpoint inhibitors, and other targeted agents. Early data demonstrate overlapping hematologic and vascular toxicities that complicate attribution and may require modified dosing strategies and more intensive monitoring. Future studies should focus on defining additive and synergistic toxicity effects, optimizing sequencing, and establishing combination-specific monitoring frameworks to maintain therapeutic efficacy while minimizing toxicity [3,26].

## 5 Limitations

Several limitations should be considered when interpreting these findings. The available evidence was limited to four randomized controlled trials, which restricts the precision of pooled estimates and reduces statistical power for infrequent but clinically important outcomes such as venous thromboembolism and grade ≥3 proteinuria. For these rare events, the resulting wide confidence intervals reflect substantial uncertainty, and pooled estimates should therefore be interpreted cautiously rather than as definitive risk estimates. Differences in treatment exposure duration and follow-up time across trials may also have influenced the observed incidence of adverse events, particularly for cumulative toxicities associated with prolonged VEGFR inhibition.

Adverse event definitions and reporting practices were not fully standardized across trials. Outcomes such as hemorrhage and dermatologic toxicity were analyzed using trial-reported CTCAE categories, which may differ in anatomic site inclusion and grading thresholds. Variability in how hemorrhage was defined or categorized across studies could influence pooled estimates of grade ≥3 bleeding, depending on whether broader bleeding categories or more restrictive definitions were applied. Baseline bleeding risk factors, including prior anticoagulant use, mucosal tumor involvement, and underlying vascular comorbidities, were not consistently reported across trials, limiting the ability to adjust for confounding. As a result, observed hemorrhage estimates may partially reflect unmeasured baseline risk rather than treatment effect alone.

The analysis of rare events introduces additional methodological challenges. Several outcomes contained zero-event cells, requiring the use of continuity correction methods. Although sensitivity analyses were conducted, such approaches can affect relative risk estimates and further limit precision for rare outcomes, particularly venous thromboembolism and high-grade proteinuria.

Inclusion of the FRUTIGA trial, which evaluated fruquintinib in combination with paclitaxel, introduces potential confounding related to combination therapy. Paclitaxel has independent vascular and renal toxicity profiles, and its concurrent use may influence adverse event rates observed in the experimental arm. Although sensitivity analyses excluding FRUTIGA did not materially alter the direction of the primary safety signals, pooled estimates may partially reflect the effects of combination therapy rather than fruquintinib monotherapy alone.

Follow-up duration across the included trials was relatively limited, which restricts the ability to evaluate delayed or cumulative toxicities associated with prolonged VEGFR inhibition. The absence of direct head-to-head randomized comparisons with other later-line therapies prevents definitive conclusions regarding the comparative safety of fruquintinib relative to alternative treatment options.

Despite these limitations, the exclusive inclusion of randomized controlled trials strengthens internal validity and reduces confounding compared with observational safety analyses. The consistent toxicity patterns observed across trials, particularly for hypertension and dermatologic toxicity, support the robustness of the principal findings and their relevance for clinical monitoring and management in contemporary gastrointestinal oncology practice.

## 6 Conclusions

This pooled analysis of randomized phase II and III trials provides an integrated assessment of the safety profile of fruquintinib across gastrointestinal malignancies. The principal toxicity signals associated with fruquintinib were grade ≥3 hypertension and dermatologic toxicity, together with increased rates of any-grade proteinuria. Grade ≥3 hemorrhage occurred at low absolute rates and was numerically higher but not statistically significant, while venous thromboembolism events were uncommon and estimates were limited by sparse event counts. These findings indicate that fruquintinib-related adverse events were observed in clinically recognizable toxicity domains, although the limited number of trials and sparse event counts for some outcomes warrant cautious interpretation.

From a clinical perspective, these results emphasize that effective toxicity monitoring and proactive supportive care are essential for maintaining treatment continuity. Early blood pressure optimization, anticipatory management of hand–foot skin reaction or palmar–plantar erythrodysesthesia (HFSR/PPE), routine urinalysis monitoring, and individualized assessment of bleeding risk may help minimize treatment interruptions and avoid unnecessary dose reductions. This approach is particularly important in later-line settings, where therapeutic options are limited and sustained exposure to active agents is critical for maintaining clinical benefit.

Continuous VEGFR inhibition remains central to the therapeutic strategy of targeting angiogenesis within the tumor microenvironment. In this context, the largely predictable and manageable toxicity profile of fruquintinib supports its continued integration into later-line and combination treatment strategies. Although hemorrhage warrants careful monitoring and patient selection, the available randomized data did not demonstrate a clear increase in VTE risk and sparse event counts and wide confidence intervals limit definitive inference.

These findings support a pragmatic clinical framework in which structured toxicity monitoring and proactive management enable sustained dosing, optimize treatment tolerability, and ultimately support the effective use of fruquintinib in patients with advanced gastrointestinal malignancies.

## Data Availability

The authors confirm that the data supporting the findings of this study are available within the article and its Supplementary Materials. All data analyzed were extracted from previously published randomized clinical trials and are available in the public domain through the original trial publications.

## Supplementary Materials

The supplementary material is available online at https://www.techscience.com/doi/10.32604/or.2026.078524/s1.
